# Mechanical Properties and Durability of CNT Cement Composites

**DOI:** 10.3390/ma7031640

**Published:** 2014-02-28

**Authors:** María del Carmen Camacho, Oscar Galao, Francisco Javier Baeza, Emilio Zornoza, Pedro Garcés

**Affiliations:** Civil Engineering Department, Universidad de Alicante, Ctra San Vicente s/n, San Vicente del Raspeig 03690, Spain; E-Mails: mc.camacho@ua.es (M.C.C.); oscar.galao@ua.es (O.G.); fj.baeza@ua.es (F.J.B.); emilio.zornoza@ua.es (E.Z.)

**Keywords:** CNT, multifunctional composites, cement, durability, mechanical properties

## Abstract

In the present paper, changes in mechanical properties of Portland cement-based mortars due to the addition of carbon nanotubes (CNT) and corrosion of embedded steel rebars in CNT cement pastes are reported. Bending strength, compression strength, porosity and density of mortars were determined and related to the CNT dosages. CNT cement paste specimens were exposed to carbonation and chloride attacks, and results on steel corrosion rate tests were related to CNT dosages. The increase in CNT content implies no significant variations of mechanical properties but higher steel corrosion intensities were observed.

## 1. Introduction

Multifunctional cement–matrix composites are useful as structural materials that provide functional properties, which allow applications such as electrical grounding, electrical contacts for cathodic protection, deicing, electromagnetic interference (EMI) shielding, antistatic flooring and strain sensing. Multifunctionality is attractive for cost reduction, durability enhancement, large functional volume, design simplification and absence of mechanical property loss (which tends to occur if embedded devices are used in place of a multifunctional structural material) [1]. Today’s demand for smart structures, capable of detecting stimulus and responding adequately, has created the need for materials with not only good mechanical properties and durability, but also new additional functions. That is the reason why many researches have been focused on the development of multifunctional materials, which combine both structural properties and other functional applications [1–11].

New functional properties include, for example, self-strain sensing [1–4], damage sensing [3,5], thermal control [6], vibration reduction [7] and electromagnetic wave reflection [8,9]. Multifunctionality is obtained by increasing the electrical conductivity of the cementitious matrix composites by adding conductive materials such as carbon or metallic admixtures. In any case, structural characteristics should be maintained or improved [5,12,13].

Carbon nanotubes (CNT) are one of the additions that can be used to create multifunctional materials. Researching and developing CNT cement matrix composites open a new line to obtain multifunctional materials, possibly more efficient and with a wider range of application fields [14,15].

Achieving good particle dispersion is the most challenging problem in the fabrication of carbon nanoparticle composites, including CNT cement based composites. CNT have unique mechanical, electrical and thermal properties [14]. However, strong van der Waals cohesive forces that occur among the fibers results in a high tendency to generate fiber clumps, which are difficult to disperse, and causes strength losses or even degradation of the original material [15]. The great advances in CNT polymer composites have not been equally achieved in cement composites, probably due to this dispersion problem.

Different methods have been used to disperse CNT or carbon nanofibers (CNF) [16], such as the combined use of surfactants and sonication, the modification of the nanotube surface or functionalizing (incorporating molecular groups to the fiber’s surface to improve the affinity with the matrix) [17,18] and even the direct growth of CNF on unhydrated cement particles [19]. Sanchez [20] studied a dry mixture of cement, CNF and silica fume, and its latter mix in water, concluding that the silica fume, due to its small particle size, helps fibers dispersion and improves interfacial interaction between the CNF and the cement hydrated phases. In another study, Yazdanbakhsh [15] reported serious difficulties, like the negative effect of using many surfactants (adequate for CNF and CNT dispersion) during the cement hydration, the CNT breaking and shortening due to an excessive ultrasonic energy, or even the deterioration of the cement-composite properties while using small particle size cements. In this research it was also concluded that a good CNT particle dispersion with surfactants in water does not mean a similar good dispersion in the paste obtain with the water mix as shown comparing optical images of the original water mix (good dispersion) with SEM images of the cement paste (poor dispersion).

Despite the potential advantages of CNT and CNF cement based composites, very few studies have been conducted in order to investigate the mechanical properties of Portland cement mortars fabricated with addition of such nanocomposites [10,14,21]. There is a wide variety of results concerning mechanical properties: examples of either huge increments [22] (even though the dispersion obtained was very poor), or no improvement at all [23] can be found. Undoubtedly, the type of CNT and its dispersion in the cement matrix must play an important role.

On the other hand, although reinforcement corrosion is probably the most important pathology in concrete, no reference to corrosion of steel rebars embedded in CNT cement composites has been found in the available scientific literature. Prior work on cement composites with addition of carbon fibers or particles showed the importance of admixtures (added to improve fiber’s dispersion) on the levels of corrosion measured, which normally were slightly higher due to the enhance composite’s conductivity [24–28]. Consequently, one of the objectives of the present research is the study of the corrosion kinetics of steel reinforcements embedded in cement pastes under aggressive conditions, such as carbonation and chloride attack. To this purpose, in order to characterize its durability, the corrosion of steel reinforced CNT cement pastes with different CNT dosages and subjected to accelerated carbonation and the action of seawater was investigated.

## 2. Results and Discussion

### 2.1. Mechanical Properties Tests in CNT Cement Mortars

**Figure 1.**
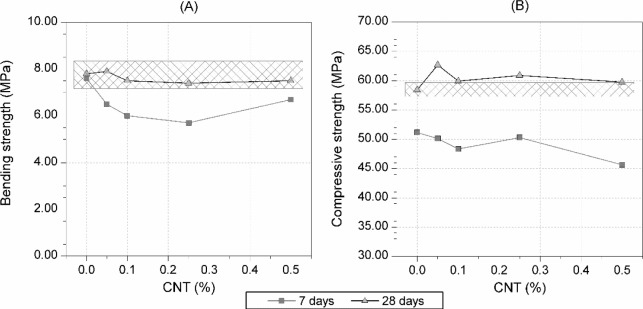
(**A**) Bending strength and (**B**) compressive strength of mortars with different dosages of CNT tested at 7 and 28 days curing time. The hash-marked area represents the confidence interval for the average value of the 28-days reference mortar without CNT, with a confidence level of 95% according to UNE 66040:2003 [29].

Figure 1 shows the bending and compressive strengths for each CNT dosage of cement mortars submerged in water for 7 and 28 days. The hash-marked area in both plots represents the confidence interval for the mean of the 28-day reference mortar without CNT, with a confidence level of 95%, assuming a normal (Gaussian) statistical distribution. Regarding the bending strength, Figure 1A, all mortars with CNT showed lower strength than the control samples, without CNT, for a 7 days curing time. The increase in CNT dosage did not significantly affect bending strength after a 28-days curing age. Figure 1B shows the influence of the quantity of CNT addition on the compressive strength, for Portland cement mortars tested at curing ages of 7 and 28 days. No significant variations can be observed due to the addition of CNT.

**Figure 2.**
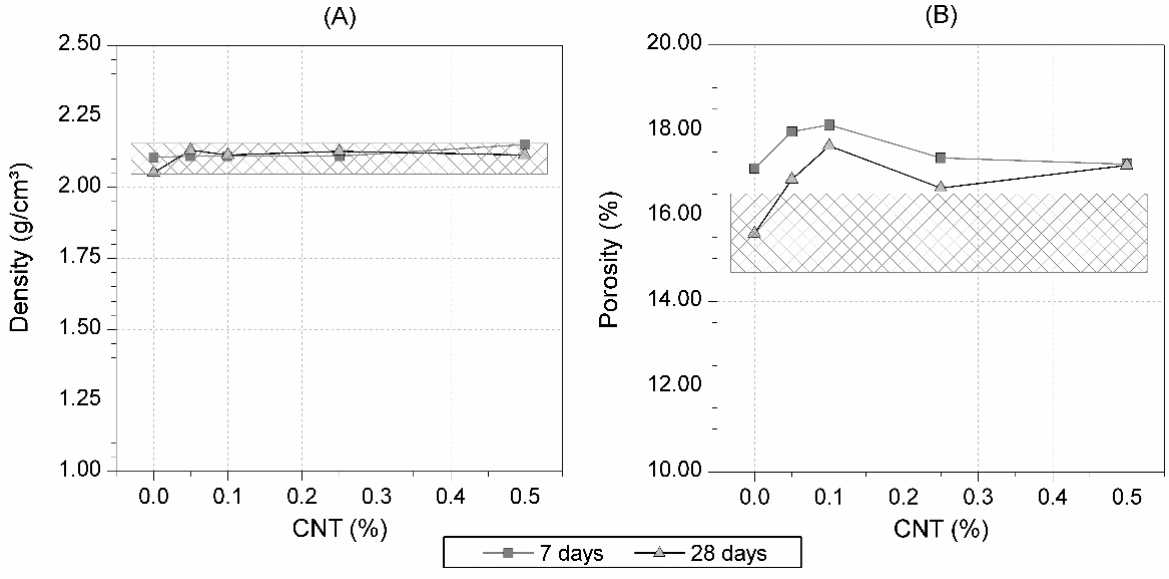
(**A**) Apparent density and (**B**) porosity of mortars with different amount of CNT tested at 7 and 28 days curing time. The hash-marked area represents the confidence interval for the average value of the 28-days reference mortar without CNT, with a confidence level of 95%, according to UNE 66040:2003 [29].

Figure 2 shows the influence of the quantity of CNT addition in the porosity and apparent density for each CNT dosage, in Portland cement mortars cured in water for 28 days, respectively. Mortar density did not significantly change with the content of CNT. However, a slight increase in sample porosity was registered due to CNT addition (between 6.8% and 13.2% with respect to samples without CNT).

### 2.2. Corrosion Tests of Embedded Steel Rebars in CNT Cement Pastes

Reinforced concrete rebars are usually passivated due to the high pH level provided by the cement hydration products. Nevertheless, some circumstances can dissolve the passive film on the steel surface. The main processes leading to the destruction of the passive film are the carbonation of concrete cover and/or the presence of chlorides over the critical concentration threshold. The consequence of transition from passive to active corrosion state is a rapid decrease of the structural element service life.

The aim of this study was the characterization of the corrosion rate observed in embedded rebars in cement pastes with addition of different dosages of CNT. The specimens have been exposed to different aggressive environments. One of these aggressive environments has been accelerated carbonation. On the other hand, the chloride ion depassivating action has been studied by partial immersion of the specimens in a solution simulating seawater.

**Figure 3.**
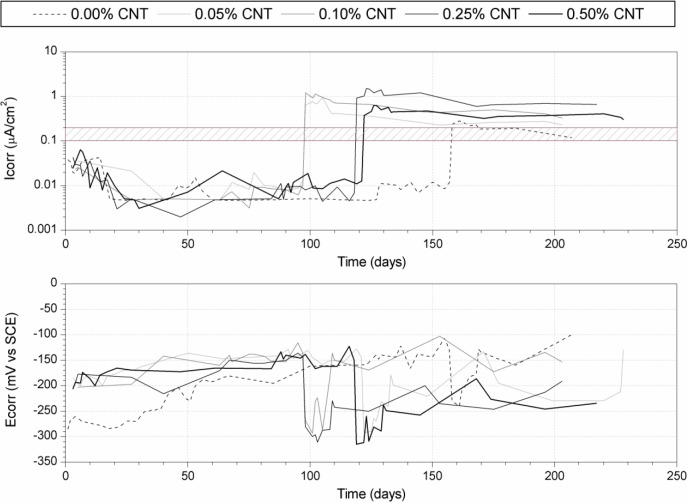
Evolution of corrosion potential (*E*_corr_) and corrosion rate (*I*_corr_) of steel rebars embedded in CNT cement pastes exposed to accelerated carbonation.

Figure 3 depicts the evolution of corrosion potential (*E*_corr_) and corrosion rate (*I*_corr_) of steel rebars embedded in CNT cement pastes, exposed to accelerated carbonation. Cement pastes have different contents of CNT: 0%, 0.05%, 0.10%, 0.25% and 0.50% with respect to cement mass. The hash-marked range in the corrosion rate between 0.1 and 0.2 μA/cm^2^ has been included, showing the threshold between active corrosion (above 0.2 μA/cm^2^) and passivity (below 0.1 μA/cm^2^) [30]. As expected, during the curing period a decreasing tendency of the steel corrosion rate is consistent with the formation of the passivating layer on the steel surface, and *I*_corr_ below the corrosion threshold. A high increase in the corrosion rate was observed as soon as the carbonation process began. This phenomenon was related to the depassivation of the steel surface due to a pH decrease because of the carbonation of the cement cover. The decrease in the corrosion potential implied the corrosion rate increase. Once the cement paste cover had been completely carbonated, no further differences in corrosion rate were observed. The increase in CNT dosages implied higher final corrosion rates.

**Figure 4.**
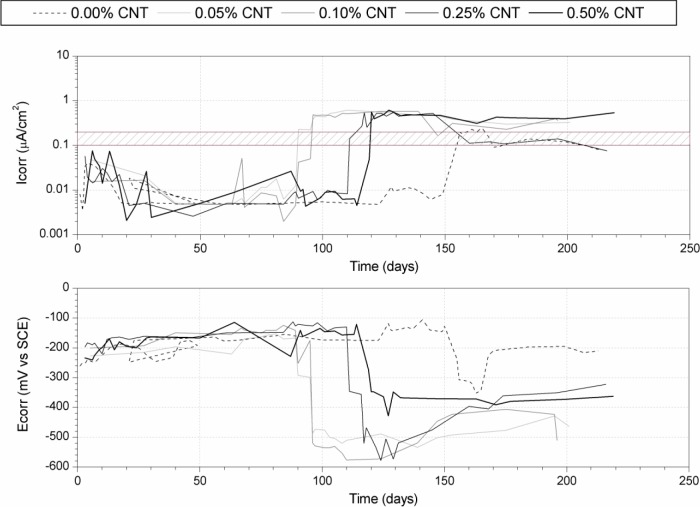
Evolution of corrosion potential (*E*_corr_) and corrosion rate (*I*_corr_) of steel rebars embedded in CNT cement pastes partially immersed in seawater.

Figure 4 shows the corrosion rate (*I*_corr_) of steel rebars embedded in CNT cement pastes partially immersed in seawater. In a first stage, before the chloride attack, the specimens were stored at 100% RH and 20 °C to obtain an adequate development of the cement matrix. As in the carbonation tests (Figure 3), a correct passivation state was achieved for all specimens after 28 days. After the curing period, the specimens were partially immersed in a 0.5 M NaCl solution simulating seawater chloride concentration. Increases in *I*_corr_ values were then observed. However, a different behavior was observed concerning the initiation period. The higher the CNT content (0.05%, 0.10%, 0.25% and 0.50%), the longer the initiation period was (80, 90, 115 and 125 days respectively), while active corrosion on the control sample was not detected until 160 days, probably due to the differences between specimens porosities. Nevertheless, once the propagation period was reached in each specimen, corrosion rate values measured were above the corrosion threshold.

Two different aspects should be considered to explain the different behavior shown by the steel rebars embedded in cement pastes with different CNT contents. On one hand, the increase in CNT addition, which is an electrical conductive material, implies a progressive decrease in the concrete electrical resistivity [5]. This fact contributes to the development of the corrosion cell, which may explain the increase of the *I*_corr_ values. On the other hand, the galvanic couple between the steel and the conductive carbon material should be taken into account. The union of two different conductive materials with different nobility implies that the less noble tends to develop higher corrosion rates than the same element without such electrical contact. On the other hand, the material with higher nobility develops lower corrosion rates. The former argument is consistent because the electrons of the less noble material (steel) will cause cathodic protection on the other one (CNT). For this reason, a higher content of CNT implies higher levels of the *I*_corr_ values.

As a consequence of these two factors, it can be expected that the higher CNT dosage would lead to higher conductivity and therefore higher galvanic couple effect from the carbon material to the steel reinforcement. Finally, the complementary action of these two factors implies higher corrosion levels, which is consistent with the obtained results.

## 3. Experimental Program and Materials

### 3.1. Materials and Sample Fabrication

Cement mortars were used for mechanical tests (compressive and bending strengths), porosity and density. Cement pastes were used for corrosion rate tests (in order to maximize the influence of the cement matrix on the corrosion process). The materials used in this research were: Portland cement type EN 197-1 CEM I 52.5 R (CEMEX España S.A., Madrid, Spain); Multiwall Carbon Nanotubes (MWCNT, BAYTUBES C 70P), supplied by Bayer MaterialScience, A.G. (Leverkusen, Germany), whose main properties are listed in Table 1; distilled water; fine aggregate, standard CEN EN 196-1 [31] silica sand (in cement mortars); Sikament FF commercial superplasticizer (SIKA, Inc., Valencia, Spain).

The water/cement ratio (w/c) for all mortars and pastes was 0.5, and the cement/sand ratio (c/s) for mortars was 1/3. CNT dosages were 0%, 0.05%, 0.1%, 0.25% and 0.5% by cement mass. The same dosages of CNT and superplasticizer were used for mortars and pastes, and were previously assessed according to Spanish Standard UNE 83258:2005 [32] in order to obtain the same workability for all mortars and pastes. Thus the quantities of plasticizer were 0%, 0.4%, 0.5%, 0.9% and 2.2% of the cement mass, for CNT dosages of 0%, 0.05%, 0.1%, 0.25% and 0.5%, respectively.

CNT dispersions for mortars and pastes were done according to a previously checked method in polymer composites [33]. CNT and distilled water were mixed in a rotatory flat-blade mixer and then treated using an ultrasound device model Hielschier UP200S. The resulting mixture was mixed with cement (and sand, in mortars) and superplasticizer in a laboratory planetary mixer for 5 min. Mortars and pastes were fabricated in laboratory conditions: 20 °C temperature and 65% relative humidity (RH). This dispersion method has been successfully used in CNF cement composites [5,10].

**Figure 5.**
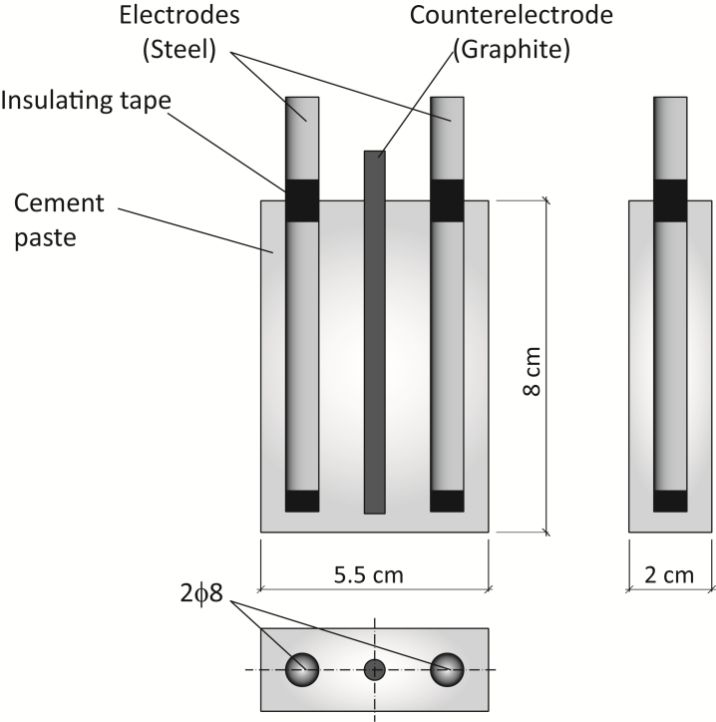
Dimensions of the specimens for corrosion tests.

Prismatic specimens of 4 × 4 × 16 cm^3^ were fabricated according to European Standard UNE EN 196-1 [31] for mechanical tests in mortars. They were cured in water and tested at 7 and 28 days curing ages. Mechanical tests were accomplished in laboratory conditions according to UNE EN 196-1 [31]. Prismatic specimens of 80 × 55 × 20 mm^3^ were prepared for corrosion rate tests. Each one contained two 8 mm diameter cylindrical steel electrodes and a graphite counterelectrode in the middle. The thickness of the resulting cement paste cover was 6 mm and the exposed steel area was 16.3 cm^2^. Figure 5 shows the specimen arrangement used for corrosion tests, similar to others used in previous works [34–38].

### 3.2. Mechanical Properties and Durability Tests

Bending and compressive strength tests on prismatic specimens were conducted according to European Standard UNE EN 196-1:2005 [31], with a ME-402/20 press machine (Servosis, S.L., Madrid, Spain). Porosity (*P*) and apparent density (*D*_ap_) were calculated after measuring dry mass (*M*_d_), submerged mass (*M*_w_) and saturated mass (*M*_s_), according to UNE-EN 993-1:1996 [39], by Equations (1) and (2). Six samples of each CNT dosage were tested:

$$p(\%)=100\times (M_{s}-M_{d})/(M_{s}-M_{w})$$ (1)

$$D_{\text{ap}}=M_{s}/(M_{s}-M_{w})$$ (2)

Corrosion rate (*I*_corr_) and corrosion potential (*E*_corr_) were measured in each steel electrode, two electrodes were measured for each specimen. Each process was long enough to obtain steady *I*_corr_ values.

**Figure 6.**
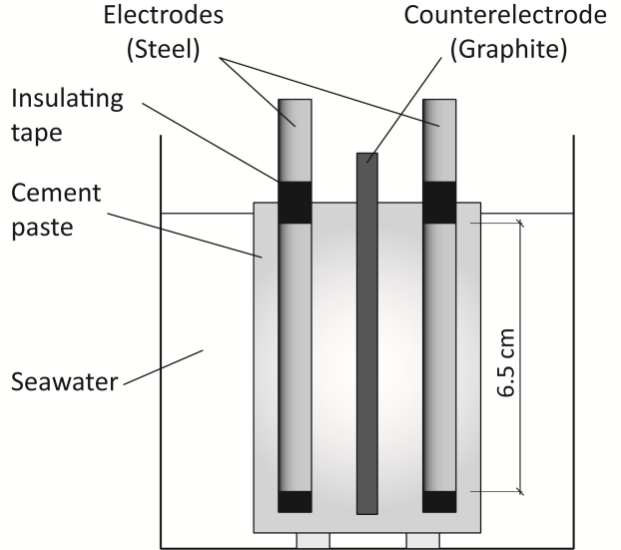
Specimen partially immersed in seawater for chloride attack tests.

After curing in ambient-controlled room (100% RH and 20 °C), one specimen of each dosage was immersed in a 0.5 M NaCl solution, simulating seawater. All these tests were done at 20 °C temperature. Some of the specimens were partially immersed in the solution, leaving 1 cm above the water level to avoid direct contact of the electrodes with the solution, as shown in Figure 6. The other samples were exposed to an accelerated carbonation process in 100% CO_2_ atmosphere and 65% ± 5% RH. Polarization resistance technique was used for testing all samples, and instant corrosion rate (*I*_corr_) was calculated using Geary and Stern equation [30]:

$$I_{\text{corr}}=B/P_{\text{r}}$$ (3)

where *I*_corr_ is the corrosion rate (μA/cm^2^); *P*_r_ is the polarization resistance (kΩ·cm^2^) and B (mV) is a constant, assumed equal to 26 mV for the steel-cement system [30]. *I*_corr_ and *E*_corr_ were periodically tested. All the potential values were referred to the saturated calomel electrode (SCE). In order to determine the polarization resistance a 362 EG&G potentiostat (Princeton Applied Research) was used.

At the end of the experiment, each steel rebar was removed and their gravimetric weight loss determined. The electrochemical weight loss values were estimated by integrating *I*_corr_ *vs*. time curves and results were compared to the corresponding gravimetric losses (obtained directly by mass differences). The good agreement between both results validates the assumed B value [30,34–38].

## 4. Conclusions

In the present research, the influence of adding CNT to cement composites was studied in two different aspects. First, some mechanical properties of CNT cement mortars, and second, corrosion rates of steel rebars embedded in CNT cement pastes, were studied. The following conclusions could be drawn.

The addition of CNT to Portland cement mortars does not significantly affect the bending strength (less than 6%) or the compressive strength (less than 7%), at 28 days curing time.

The addition of CNT to Portland cement mortars does not significantly affect the apparent density, at 28 days curing time. Only slight increases in the porosity of CNT cement mortars can be detected at the same age (between 7% and 13% with respect to the control sample without CNT).

The addition of CNT to the cement matrix could imply the development of higher levels of corrosion in aggressive conditions, such as carbonation and contamination by chloride ions.

## Figures and Tables

**Table 1.** Properties of Baytubes^®^ C 70 P Multiwall carbon nanotubes (CNT).

Properties	Value	Unit
C-Purity	>95	%
Free amorphous carbon	–	–
Outer mean diameter	~13	nm
Inner mean diameter	~4	nm
Length	>1	μm
Bulk density	45–95	kg/m^3^
Elastic modulus	3596	MPa
Tension at break	72.9	MPa
Elongation at break	10.7	%
Izod-Impact at 23 °C	103	J/m
